# EUS-guided gastroenterostomy using a parallel enteric tube for luminal distension: Prospective multicenter procedural standardization (with video)

**DOI:** 10.1097/eus.0000000000000123

**Published:** 2025-06-13

**Authors:** Carlos Chavarría, Vanessa Martín-Álvarez, Jose Ramón Aparicio, Jose Carlos Subtil, Francisco Javier Garcia-Alonso, Juan J. Vila, Belén Martinez-Moreno, Victoria Busto Bea, Carlos de la Serna-Higuera, Manuel Perez-Miranda

**Affiliations:** 1 Hospital Universitario Río Hortega, Instituto de Investigación Biomédica de Valladolid, IBioVALL, Valladolid, Spain; 2 Hospital General Universitario Dr. Balmis, Instituto de Investigación Sanitaria y Biomédica de Alicante, ISABIAL, Alicante, Spain; 3 Gastroenterology Department, Clínica Universitaria de Navarra, Navarra, Spain; 4 Gastroenterology Department, Hospital Universitario de Navarra, Pamplona, Spain.

**Keywords:** EUS-guided gastroenterostomy (EUS-GE), Gastric outlet obstruction (GOO), Lumen-apposing metal stent (LAMS), Parallel enteric tube (PET)

## Abstract

**Background and Objectives:**

The EUS-guided gastroenterostomy (EUS-GE) technique remains nonstandardized. We primarily aimed at standardizing parallel enteric tube (PET)–assisted EUS-GE, secondarily assessing reproducibility and outcomes.

**Methods:**

This prospective multicenter study included consecutive adult patients with unresectable malignant gastric outlet obstruction undergoing primary EUS-GE between August 2019 and April 2021. Hierarchical task analysis predefined procedural steps into tasks and subtasks. Subtasks were further categorized into essential (performed in all centers and in more than 85% of the procedures) or optional. Subtask methodology was considered established if performed similarly in all centers or variable if not. Procedure times, injected fluid volume, accessories, adverse events (AEs), and outcomes were recorded.

**Results:**

Seven endoscopists performed EUS-GE in 65 patients (50.8% male, median [interquartile range] age 77.5 [65.7–86.5] years). EUS-GE was categorized into 4 tasks (enteric tube placement, endoscope exchange, small bowel distention plus targeting, lumen-apposing metal stent placement) and 10 subtasks (7 essential, 3 optional). Five essential subtasks involved an established methodology (guidewire and PET placement, endoscope exchange, delivery system insertion, and lumen-apposing metal stent deployment).

Technical and clinical success rates were 98.5% and 83.3%, respectively. AEs occurred in 10 (15.4%) patients. Success and AE rates were not different between expert and nonexperts. Procedure time was longer (35 [30.6–43.7] *vs*. 21.8 [16.4–29.5] minutes, *P* < 0.001) and injected fluid volume higher (510 [439–870] *vs*. 415 [255–480] mL, *P* = 0.01) in nonexperts.

**Conclusions:**

PET-assisted EUS-GE was standardized, identifying its key steps and technique variants. PET-assisted EUS-GE appears to be a reproducible procedure among advanced endoscopists with different levels of experience.

(ClinicalTrials identification no. NCT04660695).

## Introduction

Gastric outlet obstruction (GOO) occurs in up to 20% of patients with foregut malignancies.^[1,2]^ Most patients are diagnosed at advanced stages when curative surgical treatment is not possible. Symptoms of GOO include vomiting, nausea, malnutrition, and dehydration. Relieving the mechanical obstruction and restoring oral nutrition are key treatment goals.^[3,4]^

Since 2015, EUS-guided gastroenterostomy (EUS-GE) using lumen-apposing metal stents (LAMSs) has emerged as an alternative to surgical gastrojejunostomy or enteral stent placement for malignant GOO.^[5,6]^ This procedure entails insertion of a LAMS under combined EUS and fluoroscopic guidance from the stomach into the small bowel target distal to the malignant obstruction.^[7]^

Several technical variations of EUS-GE have been described to date, largely in retrospective studies.^[8]^ Regardless of technical variations, luminal distention of the small bowel target prior to inserting the LAMS delivery system is a critical step of EUS-GE.^[9]^ Using a standard nasobiliary drain as a parallel enteric tube (PET) to deliver fluid into the small bowel distal to the obstruction prior to transgastric LAMS insertion appears simple and reproducible.^[10,11]^ However, the technical aspects of PET-assisted EUS-GE are not currently standardized. Data from prospective studies with predefined protocol and outcome parameters could help standardize the procedure. We aimed to standardize the procedural steps of PET-assisted EUS-GE in a multicenter study. As secondary aims, EUS-GE reproducibility across operators with different expertise levels, together with technical outcomes, was also assessed.

## Methods

The PENGUIN (Parallel ENteric tube-Gastroenterostomy by Ultrasound In Neoplasia) study is a multicenter, observational, prospective institutional review board–approved study conducted at 4 Spanish centers, between August 2019 and April 2021 (PENGUIN registration: NCT04660695). Written informed consent was obtained from all patients before the procedure. Clinical outcomes of this study have been reported.^[12]^

### Study population

Study population comprised consecutive adult patients with unresectable malignant GOO undergoing primary PET-assisted EUS-GE. Patients with surgically altered anatomy, prior GOO treatment (surgical gastrojejunostomy or stent placement), concurrent obstructive jaundice, evidence of secondary gastrointestinal tract strictures, grade ≥2 ascites, uncorrectable coagulopathy (international normalized ratio >1.5), severe thrombocytopenia (<50,000 platelets/μL), and failed guidewire passage were excluded from the study.

### Standardization of the procedure

A taxonomy was developed using hierarchical task analysis (HTA) to standardize PET-assisted EUS-GE. HTA is a systematic breakdown of complex procedures into sequential tasks and subtasks that has successfully been applied to various surgical procedures.^[13,14]^ HTA aims at identifying consecutive actions required during a given procedure while delineating the most challenging steps with the highest risk of repetition and/or adverse events (AEs). HTA can either be used to offer guidance to trainees and nonexperts or to objectively assess and evaluate operator technical skills.

**Figure 1:**
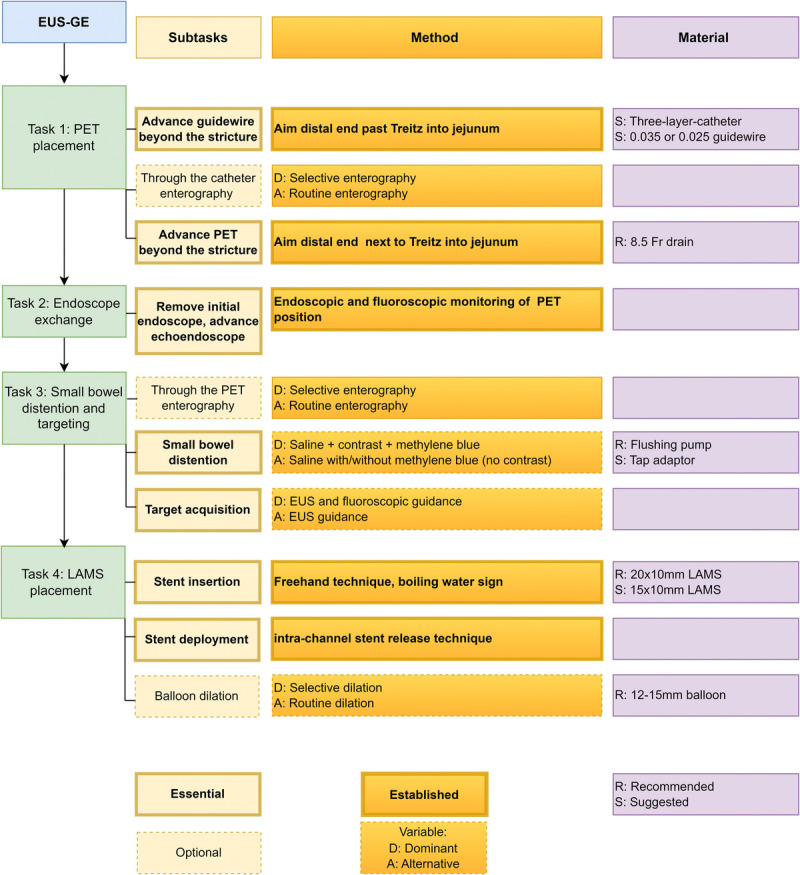
Proposed taxonomy for the PET-assisted EUS-GE with the different levels of tasks and subtasks. Subtasks are categorized into essential (performed in all centers and in over 85% of the procedures) or optional. Methodologies are categorized into established (performed similarly in all centers and in >85% of the procedures) and variable. Variable methodologies were further classified into dominant (used in ≥50% of centers and in over 40% of procedures) or alternative methodologies. The material was categorized into recommended (if >80% of endoscopists agreed) or suggested. PET: Parallel enteric tube; EUS-GE: EUS–guided gastroenterostomy; LAMS: Lumen-apposing metal stent.

Endoscopists performing EUS-GE were classified based on their prior EUS-GE experience into nonexpert (<25 procedures), proficient (25–40 procedures), and master (>40 procedures) operators, according to procedural landmarks found in a previous learning curve study.^[15]^ For dichotomous analysis, proficient and master operators were grouped together as experts. Expert operators (M.P.-M., J.R.A., J.C.S.) developed a preliminary taxonomy, based on experience gained throughout previous procedures. Following an iterative process of reevaluation, 4 sequential tasks were established, each divided into subtasks. Subtasks were further categorized into essential and optional. Subtasks performed at every participating center and in at least 85% of the procedures were defined as essential, whereas subtasks not meeting those threshold values were defined as optional. Based on the methodology used, subtasks performed using different technical variations were defined as variable, whereas those performed similarly at every center and in more than 85% of the procedures were defined as established. For variable subtasks, the variant used at ≥50% of centers and in >40% of procedures was considered the dominant method, whereas the remaining methods were labeled as alternative. Accessories used for each subtask were classified into recommended (if >80% of endoscopists agreed) or suggested (for accessories not reaching the 80% agreement threshold). The resulting taxonomy is shown in Figure 1.

### Data collection

Demographic variables and baseline clinical data were recorded at inclusion. Procedure-related data were retrieved during the endoscopic procedure by a nonoperator assistant using a predefined case report form and included the following: tasks and subtasks performed and repeated, equipment and accessories used, time intervals between procedural steps, and unplanned procedural events (UPEs), as defined by Teoh et al.^[16]^ The short and long axes of the target small-bowel loop were measured before stent placement by EUS. Regardless of local follow-up, patients underwent centralized follow-up via telephone calls by a physician or an experienced research nurse, at enrollment and at 1, 7, and 30 days after the procedure.

During the data retrieval period, several online meetings were held by the participating endoscopists. Observed differences in procedural technique were discussed. After procedure data analysis, endoscopists were queried regarding encountered differences. Endoscopist feedback from online meeting interviews was used to revise and improve the taxonomy.

### Outcomes

Technical success was defined as proper LAMS placement across the small bowel and gastric walls resulting in creation of a gastroenterostomy. This was confirmed by visualization of intestinal mucosa and/or retrograde passage of fluid from the small bowel into the stomach.

Oral intake was assessed at baseline and at days 1, 7, and 30 using the Gastric Outlet Obstruction Scoring System (GOOSS): 0 = no oral intake, 1 = liquids only, 2 = soft solids, and 3 = full diet.^[4,17]^ Clinical success was assessed in patients with technical success and was defined as a GOOSS ≥2 in the 3 days before the follow-up visit.

UPEs were considered as any deviations of EUS-GE from the planned procedural steps. UPEs included equipment malfunction or misdeployed LAMS, even if eventually proper LAMS placement within the same session effectively prevented any subsequent AE.^[16]^ AE severity was graded according to the ASGE lexicon severity grading system.^[18]^

### Sample size calculation

For standardization, considering that EUS-GE is a relatively uncommon procedure and that each center would inevitably present minor technique variations, we estimated that at least 8 procedures per center would be needed to adequately identify these expected minor variations. Based on procedure lengths reported by Jovani et al.,^[15]^ to detect a 19-minute difference in procedure time between experts and nonexperts, we estimated that at least 26 patients in each group would be required, assuming a common standard deviation (SD).

### Statistical analysis

Quantitative variables were summarized using the mean and SD or the median and interquartile range (IQR), as warranted, whereas categorical variables were described with percentages. Comparisons between continuous variables were performed using the *t* test for independent samples or the Wilcoxon rank-sum test. Categorical variables were compared using *χ*^2^ test and Fisher exact test, as warranted. Statistical significance was set at *P* < 0.05.

## Results

A total of 92 procedures were performed by 7 endoscopists from 4 different centers during the study period. As shown in Appendix I, http://links.lww.com/ENUS/A374, 65 patients met the inclusion criteria, with either concurrent obstructive jaundice (*n* = 19) or miscellaneous exclusion criteria (*n* = 8) present in the remainder. Each center included 38, 10, 10, and 7 procedures, whereas the median number of procedures/endoscopist was 7 (IQR, 5–10; range, 1–28). Three expert endoscopists (one with a previous experience of >100 EUS-GEs, the remaining 2 with >25 procedures) performed 40 procedures, whereas four nonexpert endoscopists performed 25.

### Study population

Baseline characteristics of the 65 included patients are reported elsewhere^[12]^ and summarized in Table 1. The most frequent causes of malignant GOO were pancreatic (35.4%) and gastric cancer (32.3%). The most common obstruction site was the antropyloric region and duodenal bulb (52.3%).

**Table 1:** Baseline characteristics of the patients.

Age, median (IQR), yr	77.5 (65.7–86.5)
Male sex, *n* (%)	33 (50.8)
Inpatient status	59 (90.8)
ASA classification, *n* (%)	
II/III/IV	16 (24.6)/45 (69.2)/4 (6.2)
GOOSS, *n* (%)	
0 (No oral intake)	37 (56.9)
1 (Liquid diet)	19 (29.2)
2 (Soft diet)*	9 (13.9)
Etiology of malignant GOO, *n* (%)	
Pancreatic cancer	23 (35.4)
Gastric cancer	21 (32.3)
Other	21 (32.3)
Site of the obstruction, *n* (%)	
Antrum–D1	34 (52.3)
D2	16 (24.6)
D3–D4	15 (23.1)

*Frequent vomiting.

IQR: Interquartile range; ASA: American Society of Anesthesiologists; GOOSS: Gastric Outlet Obstruction Scoring System; GOO: Gastric outlet obstruction.

### Patient setup

No center used prophylactic antibiotics systematically, and only 1 used it in >50% of cases. Spasmolytic agents (butylscopolamine) were used by all endoscopists, except one, with a median dose of 40 mg (IQR, 20–40 mg). All procedures were performed under fluoroscopy. Patient position, sedation, and orotracheal intubation (OTI) were mixed. Three (75%) centers performed EUS-GE under anesthesiologist-directed sedation with OTI, whereas the remaining center used endoscopist-directed sedation. Procedures without OTI were performed in the left lateral oblique-prone position; 3 of the 4 endoscopists performing EUS-GE in intubated patients preferred a supine position (75%); the remaining endoscopist preferred a left lateral patient position. Solid gastric contents were noted in 9 (13.9%) patients, whereas 36 (55.4%) patients presented with liquid contents that could be suctioned from the gastric lumen. Electrosurgical unit settings and fluid solutions used for bowel distention are summarized in Appendix II, http://links.lww.com/ENUS/A374.

### Task 1: Placing the enteric tube

All procedures were initiated with a large-channel endoscope, either a linear echoendoscope or a therapeutic gastroscope. Once the obstruction site was identified, a guidewire was advanced through the stricture beyond the ligament of Treitz whenever possible. Two centers favored 0.025-inch guidewires, and another 2 favored 0.035-inch guidewires. Two centers routinely used an introducing catheter (8.5F Oasis One Action Stent Introduction System, or Glotip II double-lumen catheter, Cook Medical, Winston-Salem, NC) to advance the guidewire. The remaining 2 centers preferred freehand guidewire advancement, often when traversal of the obstruction with the endoscope was feasible.

All endoscopists relied on air enterogram to confirm guidewire placement, using the guidewire trajectory within the small bowel, and the spine, as fluoroscopic references. Nevertheless, contrast enterography through the introducing catheter was performed in 29 (43.9%) patients. Only 1 operator performed contrast enterography during guidewire placement routinely. Nonexpert operators required contrast enterography during guidewire navigation more frequently than experts, 17/25 (68%) *versus* 12/40 (30%), *P* = 0.002.

All centers favored an 8.5F nasobiliary drain as PET. The only center initially using a 7F nasobiliary drain changed during the study period to 8.5F based on the online meeting feedback from study coinvestigators. All endoscopists aimed at placing the distal end of the PET in the vicinity of the Treitz angle (D4/proximal jejunum), achieving it in 58 (89.2%) procedures [Video 1]. The distal end of the PET could be advanced beyond the stricture in all cases, but in 3 (4.6%) patients, it could not surpass the proximal duodenum.

### Task 2: Endoscope removal with parallel echoendoscope intubation

**Figure 2:**
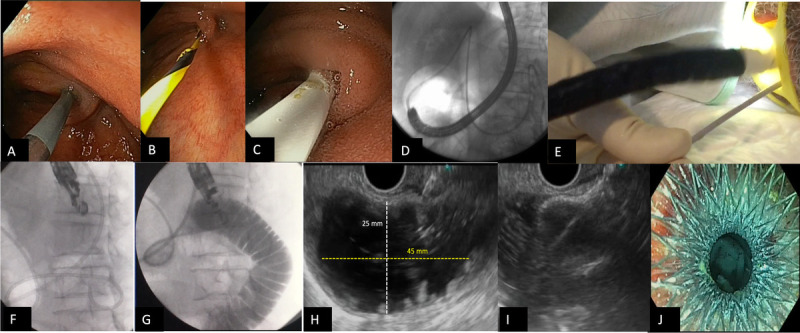
Tasks of PET-assisted EUS-guided gastroenterostomy. A–C, Task 1: Enteric tube placement. Using a large-channel gastroscope, a guidewire is passed across the stricture through a 3-layer stent-introducing catheter and advanced past the angle of Treitz into the proximal jejunum. An 8.5F PET is advanced over the guidewire through the endoscope working channel, aiming its distal end at the angle of Treitz, at the level of the spine. D and E, Task 2: Endoscope exchange. The gastroscope is removed over the PET, keeping it in position under fluoroscopy. The PET is held at the patient's mouth while the echoendoscope is advanced in parallel. F–H, Task 3: Small bowel distention and targeting. The echoendoscope position next to the PET at the angle of Treitz is confirmed under fluoroscopy before high-volume fluid injection to distend the target is begun. Access is not attempted until adequate distention is obtained, seeking both small bowel alignment with the projected LAMS catheter trajectory and an entry point perpendicular to the bowel wall. I and J, Task 4: LAMS placement. Following freehand access into the target, the LAMS distal flange is deployed under EUS, and the proximal flange is deployed within the echoendoscope working channel. After LAMS deployment, correct placement is confirmed endoscopically by identification of retrograde fluid passage through the LAMS and/or direct visualization of intestinal mucosa. PET: Parallel enteric tube; LAMS: Lumen-apposing metal stent.

Dual fluoroscopic and endoscopic monitoring of the PET position during removal of the initial endoscope was uniformly performed. The PET was then immobilized with the bite block while the echoendoscope was introduced in parallel. Three operators routinely confirmed on fluoroscopy the proximity of the echoendoscope tip to the PET past the Treitz angle before (Fig. 2F) and during (Fig. 2G) fluid injection. Transgastric EUS imaging of the pancreas body was also used as an additional landmark for the small bowel adjacent to the pancreas.

### Task 3: Small bowel distention and targeting

Bowel distention was one of the more variable subtasks. All operators sought a high-flow fluid output for adequate luminal distention and subsequent target acquisition, but the methods used and the type of fluid varied across centers. A baseline contrast enterography was performed through the PET in 24 (36.9%) cases overall. This subtask was performed at only 2 centers, with through-the-PET baseline enterogram obtained in only 20% and 57.9% of procedures, respectively.

Continuous infusion of liquid with an irrigation pump was used in 3 (75%) centers; another center connected the water tap to the PET through an in-house–made adaptor providing a flow of 600 mL/min. When the irrigation pump was unavailable, 20-mL syringes were used. The volume of fluid varied depending on the injection method; the tap adaptor method resulted in significantly higher fluid volumes than the irrigation pump 1020 (600–1200) *versus* 455 (330–555) mL, *P* = 0.001. Real-time EUS identification of small-bowel distention around the Treitz angle was sought at the beginning of injection by all operators.

Variations were similarly identified across centers in the type of injectate. Three centers used a sequential method with saline injected first and a solution of either methylene blue contrast (*n* = 2) or methylene blue only (*n* = 1) added on after the target small bowel became distended. Only warm water without any contrast or methylene blue was injected at the center using the tap adaptor method.

### Task 4: LAMS placement

The target bowel loop dimensions measured immediately before stent placement were 27 × 40 mm (IQR, 23 × 33–30 × 60 mm). Alignment between the projected LAMS trajectory and the target long axis was sought, combined with a delivery-system tip orientation perpendicular to the small bowel wall [Video 1]. All endoscopists used the freehand technique to insert the LAMS delivery system and relied on the “boiling water” sign to ascertain intraluminal access.^[19]^ The intrachannel stent release technique^[20]^ was also routinely used.

Overall, 20 × 10-mm LAMSs were used in 50 (78.1%), and 15 × 10-mm ones were placed in 14 (21.9%) patients. One endoscopist routinely utilized the 20 × 10 mm; the rest of endoscopists used 20 × 10-mm LAMS in 66.7% to 80% of cases, overall.

Balloon dilation after LAMS deployment was performed routinely in only 1 center. The rates of balloon dilation for the remaining centers were 14.3%, 18.4%, and 70%, respectively. The overall dilation rate was 38.5%. Balloon dilation was mostly performed up to 13 mm (45.8%) or 15 mm (50%).

### Postprocedure care

All centers applied a 4- to 6-hour patient monitoring interval after the procedure, before reinitiating a liquid diet. A total of 61 (95.3%) patients restarted oral feeding the day of the procedure or the following day.

### Procedure duration and outcomes

The median procedure time was 26.2 (19.7–37.1) minutes. Technical success was achieved in 64/65 patients (98.5%). In 1 case, no small bowel loops could be identified within 25 mm of the gastric wall. The baseline GOOSS (0.6 [SD, 0.73]) was significantly increased at day 7 (2 [SD, 0.75], *P* < 0.001) and at day 30 (2.3 [0.93], *P* < 0.001). Clinical success was achieved in 45 of the 54 patients (83.3%) who reached the 30-day visit, with no differences between experts and nonexperts (81.3% *vs*. 86.4, *P* = 0.46) or between 20 × 10- and 15 × 10-mm LAMS ((85.7 *vs*. 75%, *P* = 0.40).

### Unplanned procedure events and repeated subtasks

We identified UPEs during the procedure in 5 (7.7%) patients [Table 2]. A total of 10 (15.4%) patients required the repetition of at least 1 subtask (13 subtask repetitions, overall). In 7 patients, the guidewire had to be repositioned, mostly because of dislodgments while removing the introductory catheter or advancing the PET. There was also 1 false guidewire passage before PET placement and 1 PET dislodgement during endoscope removal. In 3 cases, the small bowel loop had to be distended a second time; in 2 of them, the identified target was lost, and the distention attained disappeared, whereas in 1 patient, the target could be identified again without further fluid instillation. Finally, LAMS deployment was repeated in a case following stent deployment failure, which required closure of the gastric wall with hemostatic clips and repeat access to the small bowel with a new LAMS.

**Table 2:** Subtask duration, need for repetitions, and UPEs.

Subtask	Length, min, med (IQR)	Repeated attempts, *n* (%)	UPE
Guidewire placement (T1)	4.4 (2.2–6.6)	7 (10.6)	None
Enteric tube placement (T1)	2.2 (2.2–4.4)	1 (1.5)	None
Endoscope exchange (T2)	1 (1–2.2)	1 (1.5)	None
Small bowel distention (T3)	7.7 (5.6–8.7)	2 (3)	Pump failure
Target acquisition (T3)	6.6 (4.4–10.9)	3 (4.5)	None
LAMS deployment (T4)	2.2 (1–2.2)	1 (1.5)	Electrosurgical unit failure Partial liberation failure Distal flange malposition* (*n* = 2)
Balloon dilation (T4)	4.4 (2.2–10.9)	0	None

*Two cases of type I stent misdeployment according to Ghandour et al.,^[21]^ both of them managed with a bridging stent.

IQR: Interquartile range; T: Task; UPE: Unplanned procedural event.

### Adverse events

AEs occurred in 10 patients (15.4%), as detailed in Table 3. Although most AEs were mild or moderate, there was a fatal case in a patient who developed persistent vomiting with renal and heart failure. Due to the patient's poor baseline performance status, treatment was limited to comfort measures. We observed 1 mild case of unexplained isolated fever and a case of bacteremia, both in patients who did not receive antibiotic prophylaxis.

**Table 3:** Procedure related adverse events.

Type	Day of presentation	Management	Severity
Bleeding	Day +1	Endoscopic (epinephrine injection)	Moderate
	Day +4	Conservative	Moderate
Perforation	Day +0	Conservative (IV/oral antibiotics)	Mild
	Day +0	Conservative (IV antibiotics)	Moderate
	Day +6	Conservative (IV antibiotics)	Moderate
Other			
Isolated fever	Day +0	Conservative (IV/oral antibiotics)	Mild
Aspiration pneumonia	Day +0	Conservative (IV antibiotics)	Moderate
Bacteremia	Day +1	Conservative (IV antibiotics)	Moderate
Stent dysfunction	Day +2	Endoscopic (2nd EUS-GE)	Moderate
Heart failure	Day +2	Conservative	Fatal

SEMS: Self-expandable metal stent; LAMS: lumen-apposing metal stent; EUS-GE: EUS-guided gastroenterostomy.

### Expertise and outcomes

No differences were observed between experts and nonexperts regarding technical and clinical success, AEs, or the need to repeat subtasks [Table 4]. Overall, experts required less time to perform the whole procedure compared with nonexperts (21.8 min [16.4–29.5] *vs*. 35 min [30.6–43.7], *P* < 0.001). Nonexperts took longer to identify a possible target (6.6 min [4.4–9.8] *vs*. 4.4 min [4.4–6.6 +; *P* = 0.04), to consider the target bowel loop fully distended and ready to place the LAMS (8.7 min [7.6–16.4] *vs*. 6.6 min [4.4–10.9]; *P* = 0.003), and also took a longer time to confirm proper creation of the gastroenteric fistula and to dilate the stent (8.7 min [4.4–17.5] *vs*. 2.2 min [2.2–6.6]; *P* = 0.001). Although nonexperts also required higher fluid volumes to fully distend the target bowel loop (510 [439–870] mL *vs*. 415 [255–480] mL, *P* = 0.01), the target dimensions achieved showed no differences.

**Table 4:** Influence of expertise on outcomes and procedure details.

	Overall (*n* = 65)	Experts (*n* = 40)	Nonexperts (*n* = 25)	*P*
Volume (mL), med (IQR),* mL	470 (360–600)	415 (255–480)	510 (439–870)	0.01
Target bowel loop short axis diameter, med (IQR), mm	27 (23–30)	27.5 (22.1–30)	25.2 (23.2–30)	0.72
Target bowel loop long axis diameter, med (IQR), mm	40 (33–60)	46 (33–60)	38 (31.5–51)	0.74
Procedure duration, med (IQR), min	26.2 (19.7–37.1)	21.8 (16.4–29.5)	35 (30.6–43.7)	<0.001
Repeated tasks, *n* (%)	10 (15.4)	8 (20)	2 (8)	0.29
Adverse events, *n* (%)	10 (15.4)	6 (15)	4 (16)	1
Technical success, *n* (%)	64 (98.5)	40 (100)	24 (96)	0.39
Clinical success, *n* (%)	45 (83.3)	26 (81.3)	19 (86.4)	0.72

*Procedures performed with irrigation pump.

EUS-GE: EUS gastroenterostomy; IQR: Interquartile range.

## Discussion

Various alternative techniques have been proposed for EUS-GE. The PET-assisted EUS-GE technique involves widely available through-the-scope accessories [Figure 2] and appears less challenging than EUS-GE techniques with accessories not fitting through the working channel.^[22]^ In the current study, 7 essential and 3 optional subtasks were identified within the 4 EUS-GE tasks predefined by experts through HTA and prospectively assessed in 65 procedures performed by 7 operators on naive patients with unresectable malignant GOO. Interestingly, only 5 of the essential subtasks and none of the optional ones involved an established methodology, with relative variability among the remaining subtasks.

Patient setup was similarly variable. Antibiotic prophylaxis was used routinely by only 1 endoscopist if ascites was present. Infectious AEs of EUS-GE are uncommon, and the efficacy of routine prophylaxis is unproven. Yet, cases of isolated fever and bacteremia were noted in our cohort only in patients without antibiotic prophylaxis. Butyl-scopolamine was used to minimize peristaltic movements. The only endoscopist not prescribing spasmolytics injected larger fluid volumes by the water tap method to effectively distend the small bowel. Patient position, sedation, and OTI appeared to have no influence on technical success. The left lateral decubitus was preferred by some operators because it is the standard position for upper EUS, and it was felt that fluid pooling would be favored by gravity, minimizing air artifacts. Nasogastric tube suction prior to the procedure was supported by all investigators; however, no variables potentially impacting its effectiveness (indwell time, caliber) were assessed in this study. Nonremovable gastric content was noted in more than 10% of subjects.

PET placement included 2 essential subtasks each with an established method. All endoscopists advanced the guidewire distally to the ligament of Treitz. Interestingly, this was the most repeated subtask in our series. An important clue in the placement of the 8.5F enteric tube is lodging it in the proximal jejunum next to the angle of Treitz, instead of advancing it deeper into the jejunum. This bowel segment is relatively fixed, and despite rare anatomic variations, it is usually the closest to the posterior wall of the gastric body, making it an ideal target for most malignant strictures causing GOO in patients with native upper gastrointestinal anatomy.

Keeping the enteric tube in place at the Treitz angle throughout the removal of the initial endoscope and the insertion of the echoendoscope may appear relatively cumbersome; however, it had to be repeated only once in our series (1.5%). Measures used to minimize PET dislodgement during endoscope exchange include (1) keeping the guidewire within the PET until the echoendoscope was reintroduced and (2) immobilization of the PET at the patient’s mouth by an assistant manually or by using adhesive tape to attach the PET to the patient’s bite block, or by fixing it with a mosquito clamp.

Small bowel distention and targeting were highly variable subtasks. Identifying the enteric tube on EUS as a “double rail” inside the target bowel loop has been suggested as a means to prevent accidental colonic targeting.^[21]^ However, this sign was not routinely sought by all operators or recorded in our study. Instead, fluoroscopic and EUS landmarks, together with real-time monitoring of small bowel distention, provided a reliable safeguard against this critical type of target misidentification. Continuous high-flow injection proved essential, regardless of the exact nature of the fluid administered. Our results suggest the target small bowel should be 25 × 35 mm (percentile 25 in our series) before considering bowel distention complete. Inexperienced operators might feel more comfortable with larger diameters.

All endoscopists agreed on using the freehand access and intrachannel stent release techniques for LAMS placement, concurring with previously published expert recommendations.^[23,24]^ Although all investigators used pure current surgical unit settings, 5 operators used power settings above that recommended by the manufacturer. The use of higher power generates more tissue destruction, easing stent passage through the gastric and enteric walls. Agreement was reached on the “water boiling” sign as the most accurate visual cue of the correct location of the LAMS delivery system within the enteral loop.

The 20 × 10-mm LAMS was more frequently used because of anticipated better clinical results compared with the 15 × 10-mm LAMS,^[25]^ even if this still remains to be proven. Balloon dilation post-LAMS placement was performed in selected cases to confirm proper placement and verify the absence of complications, in a minority of cases.

The technical and clinical success rates were 98.5% and 83.3%, respectively, in line with a recently published meta-analysis based on retrospective data.^[26]^ Ten AEs were reported, of which only 1 case was fatal and the other 9 were successfully treated with medical and endoscopic treatment.

To our knowledge, this is the first prospective, multicenter study on EUS-GE, conducted in a relatively short contemporary period, with endoscopists of varying levels of experience, on a homogeneous patient cohort. All procedure-related data were prospectively retrieved, using a specifically designed form. This allowed identifying the key subtasks requiring a specific methodology, while displaying the alternative methods for the remaining subtasks. However, our study was not powered to compare dichotomous variables between experts and nonexperts, or to assess the influence of the described methodological variations on clinical outcomes. In addition, 2 nonexpert investigators performed their early EUS-GE procedures under supervision, which may have influenced their results.

In conclusion, PET-assisted EUS-GE technique was standardized, with 7 essential subtasks identified, 5 of them requiring a specific methodology. PET-assisted EUS-GE thus standardized had high technical and clinical success rates with few serious AEs and could potentially facilitate the dissemination of EUS-GE.

## Supplementary Videos

Video 1. EUS-guided gastroenterostomy using a parallel enteric tube for luminal distension. Videos are available only at the official website of the journal (www.eusjournal.com).

## Source of Funding

This study has been supported by the Spanish Society of Digestive Endoscopy (SEED) and by the Gerencia Regional de Salud, Junta de Castilla y Leon GRS 1700/A/18.

## Clinical Trial Registration

PENGUIN registration: NCT04660695.

## Ethical Approval

This study was approved by institutional review board.

## Informed Consent

Written informed consent was obtained from all patients before the procedure.

## Conflicts of Interest

Carlos Chavarría received research and training support by a grant from The Foundation of the Institute of Health Sciences Studies of Castilla y León (IECSCYL) and is a consultant and speaker for Boston Scientific. José Ramón Aparicio is a consultant for Boston Scientific. Juan J. Vila is a consultant for Boston Scientific and speaker for Cook Endoscopy and Olympus. Manuel Perez-Miranda Miranda is a consultant and speaker for Boston Scientific, Olympus, and M.I. Tech. The other authors declare that they have no financial conflict of interest with regard to the content of this report.

## Author Contributions

Carlos Chavarría did study concept and design, drafting of the manuscript, and approval of the final version of the manuscript. Vanessa Martín-Álvarez, Jose Ramón Aparicio, Jose Carlos Subtil, Juan J. Vila, and Carlos de la Serna-Higuera did acquisition of data, critical revision of the manuscript for important intellectual content, and approval of the final version of the manuscript. Francisco Javier Garcia-Alonso and Manuel Perez-Miranda did study concept and design, analysis and interpretation of data, drafting of the manuscript, and approval of the final version of the manuscript. Victoria Busto Bea and Belén Martinez-Moreno did acquisition of data; administrative, technical, or material support; and approval of the final version of the manuscript.

## Data Availability Statement

The data that support the findings of this study are available from the corresponding author upon reasonable request.
